# Progress in Medicine

**Published:** 1898-10-08

**Authors:** 


### Progress in Medicine.

### DISEASES OF THE LUNGS.

(*Continued from page 9.*)

**The Open-air Treatment of Phthisis** has been receiving much attention of late, insomuch as this method, which has met with so much success abroad, is beginning to be introduced here in England. Burton-Fanning[9] says that the following are the conditions which experience has shown to be conducive to the best results in the open-air treatment of phthisis: (1) Purity of the air; this must be comparatively free from bacteria, dust, and smoke. (2) The air should be cool and bracing. (3) Abundance of sunshine is an obvious advantage, not only from its beneficial influence on the human economy, but also from the property sunlight possesses of destroying the tubercle bacillus and other microbes. (4) Dryness of the soil. (5) Very humid atmospheres are probably prejudicial to recovery from their depressing effects on the general strength, and it must be admitted that exceptional dryness characterises the air of mountain resorts; but moderate moisture is no insuperable objection, as is proved by the results obtained at Falkenstein. (6) Winds, on the whole, are unfavourable from their chilling effects. (7) Finally, elevation above the sea level is admitted to be advantageous, though, as regards healing properties, the air of altitudes differs from that of lower lying resorts only in degree and not in kind. It is very doubtful whether rarefaction of the air has any special action in phthisis. Foreign[10] sanatoria naturally fall into two groups, in one of which the patients are quartered in a single large building, whilst in the other they inhabit a number of detached cottages or villas. The first group is represented by the sanatoria at Görbersdorf, Falkenstein, Hohenhonnef, and Ruppertshain; the second by those of Nordach and Reiboldsgrüne, in Germany, and by the Adirondack Loomis Sanatoria in America. Each plan has its advantages and disadvantages. The danger which might attach to the aggregation of many consumptives is prevented by simple precautions, which are proved to be efficacious by the fact that there is not a single instance of the spread of infection from a consumptive patient in these institutions. The sputa are received into flasks or some equivalent, and spitting elsewhere is forbidden, usually on pain of instant dismissal. Linen is disinfected, and the rooms, which are specially constructed with rounded angles, are regularly cleaned with damp cloths. Carpets and hangings are absent.

The diet is very liberal; as a rule, there are five meals a day; at Nordach there are three only, but the patient is required to eat about twice as much as he feels he wants. Milk is given at all the sanatoria. Thus at Hohenhonnef three litres are taken in addition to regular meals. The views in regard to exercise differ in the various sanatoria. At Falkenstein almost perfect rest is enforced until the disease has been arrested and pyrexia entirely ceased; whilst at Görbersdorf and Nordrach moderate walking is advised to many patients who have still some degree of pyrexia. Two facts stand out clearly on comparing the treatment at these various sanatoria. At all of them a most liberal diet is provided, and secondly an absolutely open-air life is led by all the patients, whether the disease is acute or chronic, pyrexial or not in all weathers and seasons.

The two clinical results which seem constant from this open-air treatment are reduction of pyrexia and increase of appetite. It is difficult to give statistics of the results as regards cure of this open-air sanatorium treatment. Weber[11] estimates that 13 to 14 per cent. recover perfectly, 15 to 17 per cent. recover nearly perfectly, 22 per cent. remain stationary, whilst the remainder 22 to 23 per cent. become worse.

**Alpine Climates for Phthisis.**—Williams[12] says that Alpine climates are best suited for the following class of cases: The age should be under 40 years, older patients do not benefit so much. Hæmorrhagic cases as a rule do well, *i.e.*, cases where profuse hæmoptysis is associated with limited pulmonary lesions; many of these are cured by a single winter's residence. The patients should be free from pyrexia, and possess sufficient lung surface to carry on adequately the process of respiration in the attenuated atmosphere. In marked pyrexial cases the climate seems to augment the temperature.

Mountain climates are contra-indicated in the following cases: Those with double cavities and advanced stages of disease, catarrhal phthisis, laryngeal phthisis, and acute phthisis of all kinds, especially when there is great irritability of the nervous system.

Of 350 selected cases which were sent to Alpine climates by Williams, cure of the disease took place in 41 per cent., 2 per cent. remained stationary, and 14 per cent. became worse.

**Treatment of various complications of Phthisis.**—Mackenzie[13] says that in (1) fever no antipyretic drugs have any permanently beneficial effect on the course of the temperature of phthisis. (2) This fever is often accompanied by sweating at night. For this, rubbing the patient with a dry towel, the administration of food and stimulants are useful remedies. The sweating can generally be checked by drugs, of which oxide of zinc, five grains in pill form at night, is perhaps the most useful. 1-100 grain of atropine or 15 minims of tinct. of belladonna, or ½ grain of extract of nux vomica, are also excellent. (3) Hæmoptysis tends to subside of its own accord. The most important indications are quiet and rest, and a hypodermic injection of ¼ grain of morphia generally fulfils all the indications. (4) Cough. If there is active secretion from bronchi or walls of cavities expectoration is a necessity, and opiates should be avoided, and in all cases given with great care. Cough mixtures are very apt to spoil the appetite. Lozenges of gum acacia and liquorice are useful. If there is an irritable condition of the mucous membrane dry inhalations on a respirator are most useful. Twenty drops of a saturated alcoholic solution of menthol, or a similar quantity of equal parts of creosote or guiaicol and spirits of chloroform do well. When there is laryngeal catarrh a steam inhaler containing a drachm of tinct. benzoin. co. sometimes affords relief. When there is excessive secretion belladonna or codeine may be cautiously tried.

**Pneumothorax.**—Finny[14] has published an account of a remarkable case of pneumothorax which occurred in a previously healthy young man without any effusion ending in recovery, to be followed by a complete recurrence, without effusion, and again by recovery. The interval between the attacks was two months.

Pneumothorax in practically 90 per cent. of cases is the result of tuberculosis of the lung, which may be detected by physical examination. Of the remainder, a large proportion are found to subsequently develop phthisis. As a rule, effusion into the pleura follows the pneumothorax within a short time. If effusion does not follow, the prognosis, at any rate the prognosis quâ the pneumothorax is quite good, recovery seems to always occur.

The cause of these cases of pneumothorax which are not followed by effusion of fluid is very various; it may be phthisis, or emphysema, or over exertion in previously healthy and athletic persons, or injury.

[9]Burton Fanning, Practitioner, June, 1898. [10] Walters, Practitioner, June, 1898. [11] Weber, Practitioner, June, 1898. [12] Williams, Practitioner, June, 1898. [13] Mackens'e, Practitioner, June, 1898. [14] Finny, Dublin Jour. Med. Sci., April 1, 1898.

